# Initiation of antidepressants in patients infected with SARS-COV-2: Don't forget Caution for “Paradoxical” Anxiety/Jitteriness syndrome—Commentary: Prescription of selective serotonin reuptake inhibitors in COVID-19 infection needs caution

**DOI:** 10.3389/fpsyt.2023.1095244

**Published:** 2023-03-13

**Authors:** Udo Bonnet

**Affiliations:** ^1^Department of Psychiatry, Psychotherapy and Psychosomatic Medicine, Evangelisches Krankenhaus Castrop-Rauxel, Academic Teaching Hospital of the University of Duisburg/Essen, Castrop-Rauxel, Germany; ^2^Department of Psychiatry and Psychotherapy, Faculty of Medicine, Landschaftsverband Rheinland-Hospital Essen, University of Duisburg-Essen, Essen, Germany

**Keywords:** antidepressants, SNRI, SSRI, COVID-19, adverse effects, panic attacks, nausea, jitteriness

With great interest, I read the informative Opinion Article of Borovcanin et al. (1) in a recent issue of *Front. Psychiatry* about the possible benefits and obstacles (including relevant adverse effects) of selective serotonin reuptake inhibitors (SSRIs) if prescribed for patients infected with SARS-CoV-2. The SSRI fluvoxamine and further antidepressants (ADs) are probably going to be increasingly used in this particular population mainly for two reasons. First, ADs may be useful in the treatment of depression and anxiety, which are found to be frequently associated with SARS-CoV-2 infections (e.g., “coronaphobia”), COVID-19, and long/post-COVID-19 (2–5). Second, there seems emerging, albeit preliminary and still inconsistent, evidence for reducing COVID-19-related mortality and hospitalizations using a couple of ADs (5–8). This applies especially to fluvoxamine, which is, as of November 2022, the most well-studied AD in this specific research area (5–11). The cheap and easy availability of this molecule and similar ADs might facilitate an increasing prescription by physicians who may be not experienced in psychopharmacology, especially in regions where vaccination programs are still far from realization.

Therefore, in an amendment to the article by Borovcanin et al. (1), I would like to add the potential occurrence of an anxiety/jitteriness syndrome (AJS, also known as “activation syndrome”) as a common adverse reaction of ADs including SSRIs. AJS usually involves the “paradoxical” occurrence of a mild-to-severe mix of panic attacks, nausea, restlessness, insomnia, tremor, hyperhidrosis, irritability, impulsivity, and rarely also suicidality and/or hostility/aggressiveness (12, 13). AJS occurs independently of the used AD class and is one of the main causes of the early discontinuation of a selected AD (12–14). Reported incidence rates diverged considerably from 4 to 65% in persons commencing AD treatment (12–16). This large range might reflect incongruent AJS definitions [mostly symptom clusters including suicidality or not (12, 13)] as well as a variation of interlinked underlying mechanisms including individual genetic/epigenetic vulnerability, exuberant sensitization of the monoamine neurotransmitter system, and/or dis-balancing within the cytokine orchestra, as well as psychological factors [e.g., the nocebo effect of the AD treatment (12–17)]. There is no model about a possible biological mechanism of AJS being reconciled with a psychological explanation into a comprehensive explanatory model. Patients, as well as their first-degree relatives, diagnosed with anxiety and mood disorders were found to be at increased risk for AJS (odds ratio ≥ 5) (14, 15). Patients on mirtazapine were found to be at a lower AJS risk than those on other ADs (15). Another prospective study described that escitalopram, mirtazapine, milnacipran, clomipramine, and trazodone were associated with a lower incidence of AJS than paroxetine, sertraline, and fluvoxamine (14). A further study showed that high-dose AD treatment was significantly associated with AJS (15).

Usually, anxiety/jitteriness syndrome disappears spontaneously within the first weeks after its emergence, highlighting a pertinent tolerance/de-sensitization phenomenon (12–16). Although currently not proven by well-controlled clinical studies, phenothiazine-type antipsychotics (the anticholinergic activity and potential QTc prologation of which should be noted) and benzodiazepines were reported to be useful for AJS suppression (12–16) and, thereby, helpful for differentiating between AJS and a true worsening of COVID-19 or long COVID-19.

In my experience, AJS developed often immediately after starting with an SSRI or serotonin–norepinephrine reuptake inhibitor (SNRI) and disappeared rapidly within the next 2–4 days without stopping the administration of AD. Before starting with an AD, including information about AJS in the education about possibly occurring adverse events and outlining the usual transiency of AJS can stabilize the continuation of the administered AD. However, clinical studies on this specific subject are missing. Approximately two-thirds of these patients who were in the following indeed affected by an AJS continued the AD treatment in my practice using the aforementioned patient education for better clarity.

It is worth underscoring that AJS is a frequent adverse reaction in the early treatment period with an AD because this easy-to-manage, benign, and usually ephemeral condition may be overlooked if physicians are unaware of its occurrence. In this case, AJS could be more likely misdiagnosed as neuropsychiatric and/or gastrointestinal COVID-19 in patients infected with SARS-CoV-2 or worsening of pre-existing COVID-19. Nausea, tremor, anxiety, and restlessness occurring in particular within the first days after the onset of an AD treatment are more likely caused by an AJS than by COVID-19 in patients infected with SARS-CoV-2.

## Author contributions

The author confirms being the sole contributor of this work and has approved it for publication.
